# Schwann cell deletion of *Tumor Susceptibility Gene 101* ( *Tsg101* ) in mice results in severe peripheral neuropathy

**DOI:** 10.17912/micropub.biology.001406

**Published:** 2025-02-21

**Authors:** Derek Silvius, Edward Hurley, Yannick Poitelon, Kay-Uwe Wagner, M. Laura Feltri, Teresa M. Gunn

**Affiliations:** 1 McLaughlin Research Institute, Great Falls, Montana, United States; 2 Departments of Neurology and Biochemistry, Institute for Myelin and Glia Exploration, Jacobs School of Medicine and Biomedical Sciences, University at Buffalo, State University of New York, Buffalo, New York, United States; 3 Albany Medical College, Albany, New York, United States; 4 Department of Oncology and Center for Molecular Medicine and Genetics, Wayne State University, Detroit, Michigan, United States; 5 Touro University College of Osteopathic Medicine - Montana, Great Falls, Montana, United States

## Abstract

Myelinating Schwann cells are particularly susceptible to defects in endosomal trafficking. TSG101 is a component of the endosomal trafficking machinery that mediates the sorting of ubiquitinated receptors into multivesicular bodies. We previously demonstrated that deleting *Tsg101* from mouse oligodendrocytes in the central nervous system causes rapid onset de/dys-myelination and vacuolation of white matter, suggesting an important role for TSG101-dependent trafficking in myelination. Here, we show that TSG101 is also required for normal myelination in the peripheral nervous system.

**Figure 1. Histopathology of sciatic nerves lacking TSG101 in Schwann cells.**
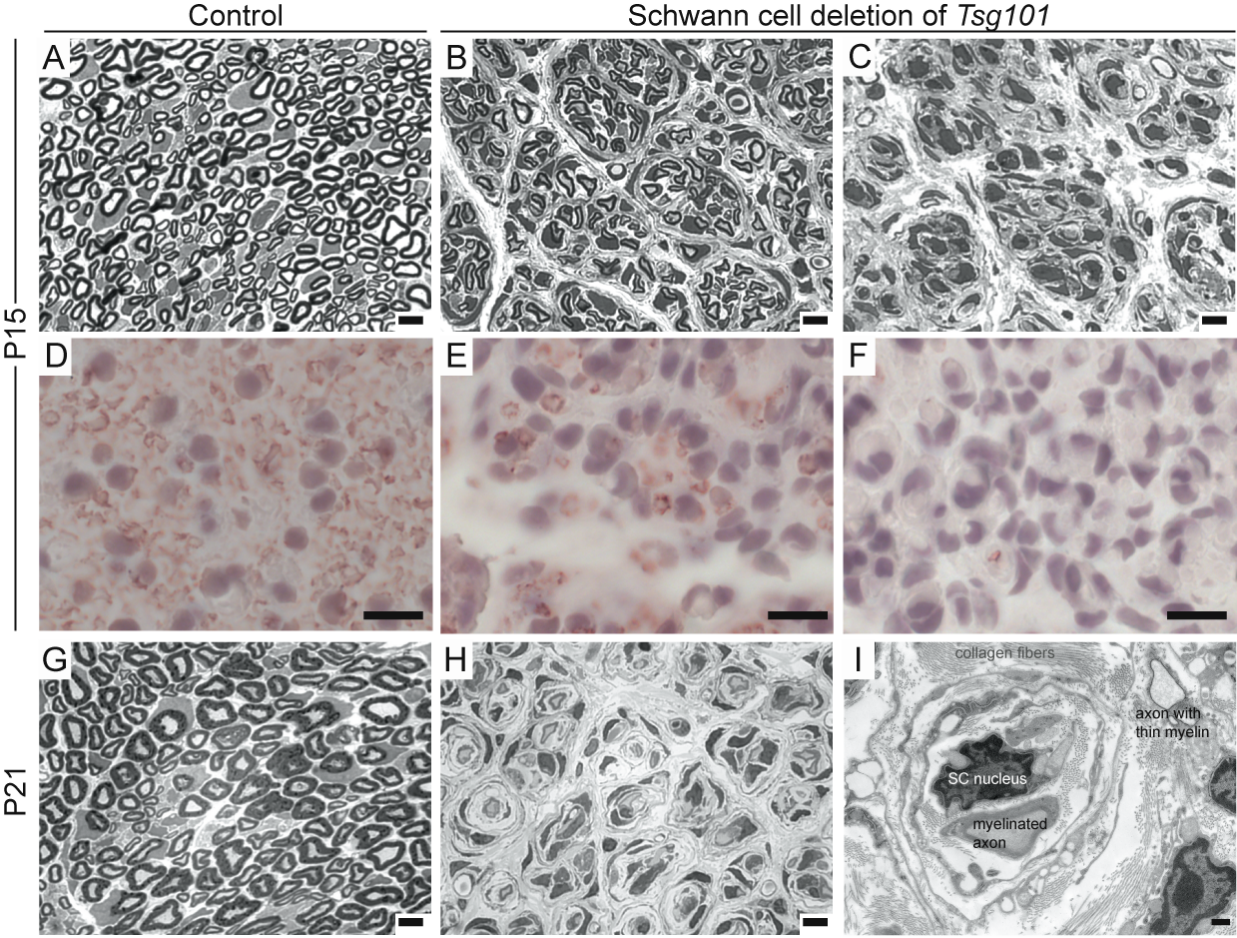
(A-C) Representative semi-thin cross sections of sciatic nerves from one control ( *Tsg101 ^fl/+^ ; P0-Cre+* ) and two different *Tsg101 ^SC-null^* (Tsg101 ^fl/fl^ ; P0-Cre+) postnatal day 15 (P15) pups showing reduction of myelin and presence of onion bulb structures in nerves from *Tsg101 ^SC-null^* mice. Scale bars: 5µm. (D-F) Representative IHC for myelin basic protein (MBP) on 7m paraffin-embedded cross sections on contralateral nerves from animals shown in A-C (B and E from same animal, C and F from same animal). Sections are counterstained with hematoxylin. Scale bars: 10µm. Note the difference in severity of myelination defects between the two *Tsg101 ^SC-null^* animals. Sciatic nerves from 5 *Tsg101 ^SC-null^* mice were examined, the images shown represent the spectrum of the phenotypes observed. (G-H) Representative semi-thin cross sections of sciatic nerves of 1 control and 1 *Tsg101 ^SC-null^* mouse at P21 showing similar but more severe myelination defects compared to P15 nerves. Scale bars: 5µm. (I) Electron micrograph (4800X) of sciatic nerve cross-section from a P21 *Tsg101 ^SC-null^* mouse. Scale bar: 500nm. Note Schwann cell layers adjacent to nucleus, indicating multiple rounds of de- and remyelination.

## Description

The endosomal pathway traffics receptor proteins and lipids into early endosomes and directs them either to recycling endosomes for trafficking back to the cell membrane or sorts them into intraluminal vesicles (ILVs) within multivesicular bodies (MVBs), which fuse with lysosomes to degrade their contents or with the plasma membrane to secrete ILVs as exosomes (reviewed in (Scott et al., 2014) ). The timing of these events is important to cellular processes since receptors can continue to activate downstream signaling pathways while they remain on early endosomes. Several forms of demyelinating CMT are caused by mutations in genes encoding proteins involved in membrane dynamics and endosomal trafficking, including *N-myc downstream regulated gene 1* ( *NDRG1), myotubularin related protein 2* ( *MTMR2), SET binding factor 2 (SBF2/MTMR13),SH3 domain and tetratricopeptide repeats 2* ( *SH3TC2), dynamin 2 (DNM2), FIG4 phosphoinositide 5-phosphatase* ( *FIG* 4), and *LPS-induced TN factor* ( *LITAF/SIMPLE* ) (reviewed in (Markworth et al., 2021) ). For example, mutations in *SH3TC2* that cause CMT type 4C cause impaired recycling of membrane components necessary for Schwann cell function (Roberts et al., 2010; Stendel et al., 2010) . Furthermore, mouse models with Schwann cell-specific deletion of *Fig4* , *HGF-regulated tyrosine kinase substrate* ( *Hgs/Hrs), or phosphatidylinositol 3-kinase catalytic subunit type 3* ( *Pik3c3)* show mild peripheral hypo/de/dys-myelination associated with altered ERBB2/3 signaling (Logan et al., 2017; McLean et al., 2022; Vaccari et al., 2015) . In Schwann cells, neuregulin 1 (NRG1) signaling through erb-b2 receptor tyrosine kinases 2 and 3 (ERBB2/B3) mediates myelination, and endosomal trafficking of NRG1-bound ERBB2/B3 regulates receptor down-regulation and recycling (Newbern and Birchmeier, 2010; Salzer, 2015) . Disrupted endosomal sorting can result in sustained activation of downstream pathways, including the ERK1/2 signaling cascade, to negatively impact Schwann cell myelin integrity (Newbern and Birchmeier, 2010; Salzer, 2015) . Thus, there is support for the idea that endosomal trafficking defects can cause Schwann cell dysfunction and demyelination, reinforcing the relevance of this pathway to myelination.

Tumor susceptibility gene 101 (TSG101) encodes a component of the E ndosomal S orting C omplex R equired for T ransport-1 (ESCRT-I), which helps mediate the sorting of ubiquitinated receptors onto ILVs of MVBs. The ESCRT-0 protein, HGF-regulated tyrosine kinase substrate, HGS (formerly referred to as HRS) mediates the initial recruitment of ESCRT-I to endosomes (via interaction with TSG101), but in multiple mammalian cell lines, siRNA depletion of TSG101 or HGS had significantly different effects on endosomal and MVB morphology (Bache et al., 2003; Lu et al., 2003; Raiborg et al., 2008; Razi and Futter, 2006) . Specifically, knockdown of TSG101 inhibited epidermal growth factor degradation and MVB formation and caused tubulation of the vacuolar domains of early endosomes, while depletion of HGS had only a modest effect on EGF degradation, did not induce tubulation of early endosomes, and resulted in the production of enlarged MVBs containing few ILVs but that could still fuse with the lysosome (Bishop et al., 2002; Doyotte et al., 2005; Razi and Futter, 2006) . These data suggest that HGS and TSG101 have distinct roles in the endosomal trafficking pathway, with TSG101 being required for the formation of stable vacuolar domains within the early endosome that subsequently develop into MVBs and HGS being more important in the formation and/or accumulation of ILVs within MVBs. Mice lacking HGS in Schwann cells developed mild motor and sensory defects, a reduced number of myelinated axons and thinner myelin sheaths in the sciatic nerve, as well as aberrantly folded myelin sheaths (McLean et al., 2022) .

TSG101 and HGS also both interact and partially colocalize with LITAF (Lee et al., 2011) , which is expressed in Schwann cells and mutations in it cause dominant demyelinating peripheral neuropathy, CMT1C (Bennett et al., 2004) . Although one study showed that CMT1C-associated *LITAF* mutations did not effect on its subcellular localization or association with TSG101 (Shirk et al., 2005) , another study showed a dominant negative effect on EGFR degradation and lysosomal trafficking of EGF, associated with reduced membrane association of HGS and TSG101 (Lee et al., 2011) . In the latter study, expression of CMT1C-associated LITAF mutants in Schwann cells caused prolonged activation of ERK1/2 signaling, presumably downstream of NRG1-ERBB2/3 signaling.

Deleting *Tsg101* from oligodendroglia in the central nervous system resulted in severe, rapid-onset myelination defects and vacuolation (Walker et al., 2016) , suggesting an important role for TSG101-dependent trafficking in signaling pathways that regulate myelination. To test whether TSG101 is also required for normal myelination in the peripheral nervous system, we investigated the consequences of deleting *Tsg101* in Schwann cells. We predicted this would cause a more severe peripheral neuropathy than deleting *Hgs,* given the stronger effect of *Tsg101* depletion on endosomal/MVB phenotypes in cultured cells.

*Tsg101* conditional knockout mice ( *Tsg101 ^tm1KuW^* , referred to here as *Tsg101 ^fl^* ) were mated to *P0-Cre* transgenic mice, which express cre recombinase specifically in Schwann cells starting on embryonic day 13.5. Cre-positive *Tsg101 ^fl/+^* offspring were backcrossed to *Tsg101 ^fl/fl^* animals. All pups that were homozygous for the *Tsg101* conditional allele and carried the *P0-Cre* transgene (referred to herein as *Tsg101* -Schwann cell null, or *Tsg101 ^SC-null^* animals) were smaller than their littermates, developed a tremor by 12 days of age, had abnormal posture of their fore- and hind-limbs (arthrogryposis), and failed to thrive. They died throughout the postnatal period, with very few surviving to 3 weeks of age. We recorded 89 affected animals out of 427 pups born from 15 different breeder pairs, for a frequency of ~21% affected. This is significantly different from the 25% expected (χ ^2^ = 4.04, p = 0.044) and is likely due to the loss of some affected animals prior to them being observed and recorded.

Histological analysis of the sciatic nerves of *Tsg101 ^SC-null^* animals at postnatal days 15 and 21 revealed striking de- and dys-myelination ( Fig. 1 ). Toluidine blue-stained semi-thin cross sections showed reduced myelin, enlargement of the interstitial space, and presence of onion bulb structures in the sciatic nerves of *Tsg101 ^SC-null^* mice by postnatal day 15 (P15, Fig. 1A-C ). The sciatic nerves of some *Tsg101 ^SC-null^* animals had fewer, thinner myelin sheaths, while others showed an almost complete absence of myelin, as detected by toluidine blue staining on semithin sections ( Fig. 1A-C ) and immunohistochemistry for myelin basic protein (MBP) on paraffin sections ( Fig. 1D-F ). The myelination defects associated with loss of TSG101 were progressive, with the sciatic nerve of a P21 *Tsg101 ^SC-null^* animal showing severe hypomyelination and presence of onion bulb formations, (Fig. G-H). Ultrathin electron micrograph (EM) analysis of P21 sciatic nerve cross-sections revealed axons with thin myelin sheaths and multiple cell layers adjacent to nucleus of Schwann cells. These “onion bulbs” comprise concentric layers of Schwann cell processes and connective tissue (collagen) arranged around axons and are consistent with multiple rounds of de- and remyelination (Iwata et al., 1998; Tracy et al., 2019; Webster et al., 1967) . This suggests that TSG101 is not essential for myelin production, but it is required for the maintenance of stable myelin sheaths. Schwann cell nuclei were still present by P21 and did not appear pyknotic ( Fig. 1F ), suggesting that loss of TSG101 did not disrupt myelination by triggering Schwann cell apoptosis.

As predicted, based on the differences observed in cellular phenotypes and EGF/EGFR degradation when TSG101 or HGS was depleted from mammalian cells by siRNA, the phenotype of *Tsg101 ^SC-null^* mice was more severe than that observed in *Hgs ^SC-null^* mice. Onion bulb formations are characteristic features in CMT1A and other demyelinating CMTs. Since TSG101 and ESCRT proteins are critical for endosomal sorting, their dysfunction could impair myelin production and turnover, exacerbating the cycles of demyelination and remyelination that contribute to onion bulb pathology. In the future, proteomic studies may shed light on the specific signaling pathways disrupted in *Tsg101 ^SC-null^* that contribute to the severe peripheral neuropathy phenotype. The variable expressivity of myelination defects and survival of *Tsg101 ^SC-null^* mice likely reflects their mixed genetic background (129S1/Sv x C57BL/6) and an effect of modifier genes, although inter-animal differences in P0-cre expression and *Tsg101* deletion cannot be ruled out. Identifying the genes and pathways that influence disease severity in these mice could reveal druggable targets to treat some forms of CMT or other peripheral neuropathies.

## Methods

*Animals*

All studies were approved by the McLaughlin Research Institute’s Institutional Animal Care and Use Committee and adhered to the Association for Assessment and Accreditation of Laboratory Animal Care guidelines. Mice homozygous for a *Tsg101* conditional knockout allele (129X1/SvJ- *Tsg101 ^tm1Kuw^* , referred to herein as *Tsg101 ^fl^* ; RRID: MMRRC_037407-MU), in which exon 1 is flanked by a floxed neo cassette inserted approximately 3 kb upstream and a single *loxP* site inserted 230 bp into intron 1 (Wagner et al., 2003) , were mated to mice hemizygous for Tg(Mpz-cre)26Mes/J (obtained from the Jackson Laboratory and referred to herein as P0-cre; RRID: IMSR_JAX:017928). *P0-cre* transgenic mice express cre recombinase in Schwann cells, under the control of the myelin protein zero (P _0_ , *Mpz* ) promoter, by embryonic day 14 (Feltri et al., 1999) . *Tsg101 ^fl/+^ ; P0-cre+* F1 offspring were backcrossed to *Tsg101 ^fl/fl^* mice and *Tsg101 ^fl/fl^ ; P0-cre+* and control ( *Tsg101 ^fl/+^ ; P0-cre+* and *Tsg101 ^fl/fl^ ; P0-cre* - *neg* ) pups were obtained from intercrossing N1 or N1F1 Tsg101fl/+; P0-cre+ x *Tsg101 ^fl/fl^ ; P0-cre-neg* sibs.

*Histology*

Sciatic nerve segments were sampled from approximately the same position for each animal. Contralateral nerves were either fixed in 4% buffered paraformaldehyde, for immunohistochemistry (IHC), or 2% buffered glutaraldehyde with postfixation in 1% osmium tetroxide (for semi- and ultra-thin sections).

For IHC, specimens from five *Tsg101 ^SC-null^* and four control animals were embedded in paraffin following standard protocols and sectioned at 5 microns. Following deparaffinization and rehydration, sections were permeabilized in a 0.2% Triton-X100 solution in PBS, then subjected to antigen retrieval in 10mM sodium citrate (pH 6.0, 100C, 10 minutes). Slides were blocked in 10% serum, then incubated with an antibody against myelin basic protein (MBP; Covance Cat# SMI-99, RRID:AB_2314772) at 1:1000, followed by horseradish peroxidase conjugated anti-mouse secondary antibody (BD Pharmingen horseradish peroxidase-conjugated anti-mouse Ig, cat# 554002, RRID: AB_395198) at 1:100 and chromogenic visualization using NovaRed substrate (Vector Labs, CA). Slides were counterstained with hematoxylin prior to coverslipping, and then examined on a Zeiss AxioImagerM1 light microscope.

Routine semithin and ultrathin (electron micrograph [EM]) analyses were performed as described (Quattrini et al., 1996) on sciatic nerves from *Tsg101 ^SC-null^* animals (n=2 at P15, n=1 at P21) and controls (n=1-2 at each age). Briefly, sciatic nerves were fixed in 2% buffered glutaraldehyde, then postfixed in 1% osmium tetroxide. After alcohol dehydration, nerves were submerged in propylene oxide, and then in a 1:1 mixture of Epon-propylene oxide. Nerves were embedded in 100% Epon, and resin was allowed to polymerize. Semithin transverse sections were sliced 0.5-μm-thick using Leica UC7, stained with 2% toluidine blue, and then examined by light microscopy with Leica DM6000B. EM transverse sections were sliced 700–900 Å-thick using Leica UC7, stained with uranyl acetate and lead citrate, and then examined with an electron microscope (model FEI BioTwin). Analyzed sections were sliced from the distal end of embedded sciatic nerve. Images acquired from semithins and EMs were nonoverlapping and comprehensive.
