# Corrigendum: Schwann cell deletion of *Tumor Susceptibility Gene 101* ( *Tsg101* ) in mice results in severe peripheral neuropathy

**DOI:** 10.17912/micropub.biology.001998

**Published:** 2025-12-22

## Description

The author has requested to add support from the National Institute Of General Medical Sciences of the National Institutes of Health under Award Number P20GM152335. The content of this article is solely the responsibility of the authors and does not necessarily represent the official views of the National Institutes of Health.

The following was added to the funding section of the published article.

"Institutional support is provided to T.M.G. through a NIH Center grant from National Institute of General Medical Sciences (NIGMS; P20GM152335)."

